# Draft Genome Sequence of a Helicobacter pylori Strain Isolated from a Patient with Diffuse Gastritis from a Region of High Cancer Risk in Colombia

**DOI:** 10.1128/genomeA.00244-15

**Published:** 2015-04-09

**Authors:** Andrés J. Gutiérrez-Escobar, Martin Bayona Rojas, Carlos Barragán Vidal, Clara Esperanza Trujillo, María Mercedes Bravo

**Affiliations:** a Grupo de Investigaciones Biomédicas y Genética Humana Aplicada (GIBGA), Universidad de Ciencias Aplicadas y Ambientales (UDCA), Bogotá, Colombia; b Grupo de Investigación en Cáncer y Agentes Infecciosos, Instituto Nacional de Cancerología, Bogotá, Colombia

## Abstract

The draft genome sequence of one Colombian Helicobacter pylori strain is presented. This strain was isolated from a patient with diffuse gastritis from Tibaná, Boyacá, a region with high gastric cancer risk.

## GENOME ANNOUNCEMENT

Helicobacter pylori is a Gram-negative microaerophilic bacterium. It has been estimated that it colonizes half of the human population (1, 2). The prevalence of the infection correlates with the socioeconomic status of the populations studied, more frequently affecting developing rather than developed countries (3). The infection is associated with the development of chronic gastritis, gastric ulcer, and stomach cancer (4), although in most cases the infection persists in the gastric mucosa without affecting the individual’s health (5). In this communication, we present the genome sequence of the strain Col2025. This strain was isolated from a 36-year-old man diagnosed with diffuse antral gastritis from Tibaná, Boyacá, a region with high gastric cancer risk in Colombia (6). Interestingly, neither CagA phosphorylation nor interleukin 8 (IL-8) secretion was detected when the strain Col2025 was cultured with AGS cells.

To obtain a genome sequence, 1 µg of DNA was fragmented by Covaris, and the fragments were repaired by attaching an A to the 3′ end. Illumina adaptors were ligated to the fragments and selected for sizes between 400 and 500 bp. The size-selected fragments were amplified by PCR and the final products validated in an Agilent Bioanalyzer. The short reads were assembled using SOAPdenovo (7). Finally, the sequences were annotated in GenBank using the NCBI Prokaryotic Genomes Automatic Annotation Pipeline (PGAAP) according to the submission guidelines (http://www.ncbi.nlm.nih.gov/genbank/genomesubmit.html). Overall, this whole-genome shotgun sequencing revealed the following data: scaffolds, 54; contigs, 73; *N*_50_, 60,455; *L*_50_, 9; genome size, 1.64; GC content, 38.9%; proteins, 1,451; rRNAs, 3; tRNAs, 36; genes, 1,543; pseudogenes, 52. This genome and others sequenced using strains isolated in Colombia (8, 9) will be the basis for evolutionary computational studies.

### Nucleotide sequence accession number.

This whole-genome shotgun project has been deposited in DDBJ/ENA/GenBank under the accession no. JOKW00000000.
